# Suicide attempts and eating disorders in adolescents, the mental health wave of the second year of the COVID-19 pandemic: A paediatric emergency department perspective

**DOI:** 10.3389/fped.2023.1078274

**Published:** 2023-01-25

**Authors:** Giorgio Cozzi, Alberto Grillone, Elettra Zuliani, Manuela Giangreco, Chiara Zanchi, Giuseppe Abbracciavento, Egidio Barbi, Alessandro Amaddeo

**Affiliations:** ^1^ Institute for Maternal and Child Health IRCCS Burlo Garofolo, Trieste, Italy; ^2^ University of Trieste, Trieste, Italy

**Keywords:** adolescence, COVID-19, eating disorders, emergency department, mental health, suicide

## Abstract

**Aim:**

We compared adolescents’ visits to a tertiary-level paediatric emergency department (PED) in Italy during the pre-pandemic year and the first and second years of the COVID-19 pandemic, focusing on mental health presentations.

**Methods:**

This was a retrospective study. We collected the number of visits, the demographical features, triage codes, discharge diagnoses, and outcomes of adolescents 13–17 years old who accessed the PED from 1 March 2019 to 28 February 2022.

**Results:**

During the study period, 13,410 adolescents accessed the PED. The number of visits related to mental health problems was 304 (6.4%) in the second year of the pandemic and 306 (5.6%) in the pre-pandemic year, *p* = 0.07. In the same periods, females’ prevalence was higher, 220 (72.4%) vs. 197 (64.4%), *p* = 0.03. The absolute number of subjects needing admission increased, 44 vs. 34, *p* = 0.21, and more urgent psychiatric consultations were needed, 161 vs. 114, *p* < 0.0001. The number of suicide attempts was 23 vs. 8, +188%, *p* = 0.01. The number of adolescents with eating disorders was 21 vs. 5, +320%, *p* = 0.001.

**Conclusion:**

PED visits for suicide attempts and eating disorders in adolescents sharply increased in the second year of the pandemic.

## Background

In the decade before the COVID-19 pandemic, a significant increase in the number of adolescents presenting to the emergency department with mental health problems was reported in Western Countries (1, 2).

It was estimated that nearly 20% of adolescents had symptoms related to a mental health disorder (3). The prevalence of these disorders was so high among adolescents that some Authors described this phenomenon as a real mental health crisis (4).

During the COVID-19 pandemic, adolescents worldwide went through periods of forced isolation at home, school closure, and bans from playing sports, social activities, and gatherings. Many young people experienced socioeconomic stress, grief for loved ones, and loss of supportive environments. In this context, several systematic reviews and meta-analyses reported rising anxiety, depressive and post-traumatic symptoms in children and adolescents (5, 6).

Therefore, we wanted to investigate the association between the pandemic and the PED flow of subjects with mental health symptoms in order to understand the possible influence of this impressive event on a population suitable to develop mental health symptoms. With this purpose, we designed a retrospective study to describe the emergency department visits of adolescents in our Institution in the last pre-pandemic year compared to the first and second year of the COVID-19 pandemic, with a particular focus on mental health presentations.

## Patients and methods

We conducted a retrospective study analyzing the medical records of all the adolescents from 13 to 17 years of age who accessed the paediatric emergency department (PED) of the tertiary level, university teaching, children's hospital, Institute for Maternal and Child Health.

IRCCS Burlo Garofolo, Trieste, Italy. Trieste is a city of 200,000 inhabitants located in the Friuli Venezia Giulia region of northeastern Italy. The Institute has the only PED in the city and each year it used to provide assistance to approximately 25,000 patients from 0 to 17 years of age in the pre-pandemic years. The Institutional Review Board of the Institute provided ethical approval for the study protocol (RC 49/2022). According to the Research Institute's policy, only patients whose parents signed the informed consent were they agreed that “clinical data may be used for clinical research purposes, epidemiology, the study of pathologies and training, with the objective to improve knowledge, care and prevention”, were included in the study.

The first case of SARS-CoV-2 infection was registered in Italy at the end of February 2020. Therefore, we decided to compare the number of visits, and their main characteristics, in the following three periods, the pre-pandemic year, from 1 March 2019 to 28 February 2020, the first pandemic year, from 1 March 2020 to 28 February 2021, and the second pandemic year, from 1 March 2021 to 28 February 2022. The data were collected from the PED's electronic database. For each assessed patient, we collected: the date of the visit, the patient's demographics, the nursing triage code, and the discharge diagnosis. Data on the need for hospital admission and the destination ward were also collected. The number of urgent psychiatric consultations in the three periods of time was also recorded. Our Institution's triage system consists of four priority levels of increasing severity: not urgent, minor urgency, urgent and emergencies and resuscitation. We categorized the diagnoses into injuries, infectious diseases, mental health problems, surgical diseases, neurological diseases, and other diagnoses.

The primary outcome of the study was to compare the number of PED visits for mental health problems in the second year of the pandemic and the pre-pandemic year.

Secondary outcomes were: to describe the prevalence of visits related to the different mental health problems and to alcohol and substance abuse in these three periods of time; to evaluate the prevalence of admissions for mental health problems in the three periods of time, to compare the extent of the variation in primary PED diagnoses across the three time periods.

### Statistical analysis

Absolute frequencies and percentages were used to describe categorical variables while median and interquartile ranges were calculated for continuous variables. The chi-square test was applied to evaluate the differences in the number of visits between the pre-pandemic year (March 2019 to February 2020), the first pandemic year (March 2020 to February 2021) and the second pandemic year (March 2021 to February 2022). Associations between categorical variables, namely triage codes, reasons for visits, diagnoses and outcomes, in the three periods were verified by Fisher's exact test or chi-square test, as appropriate. The variations in the absolute number of visits between the two study periods were reported as percentages. A *p* value of <0.05 was considered significant. All the analyses were conducted with SAS software, Version 9.4 (SAS Institute Inc., Cary, NC, USA).

## Results

During the study period, 13,410 adolescents accessed the PED. In the first year of the pandemic, we experienced a significant decrease in the number of adolescents accessing the PED compared to the pre-pandemic year: 3,163 vs. 5,501, −43%, *p* < 0.0001. On the other hand, in the second year of the pandemic, this difference was numerically less pronounced, 4,746 vs. 5,501, −14%, even if still statistically significant, *p* < 0.0001. Over the years, the distribution of the age and gender of patients remained substantially stable. The number of visits changed mainly for non-urgent visits and minor urgencies. In the second pandemic year, the prevalence of urgent and emergent cases was similar to the pre-pandemic year, 494 (10.4%) vs. 565 (10.3%) respectively.

The Table 1 shows the distribution of the primary PED discharge diagnoses in the study period. In the first pandemic year, the visits related to injuries, infectious diseases, and mental health problems considerably dropped compared to the previous year, −48%, −39%, −44%, respectively. On the contrary, in the second pandemic year, injuries and mental health problems returned to values similar to the pre-pandemic year. In particular, the number of visits related to mental health problems was the same, 304 vs. 306 visits. Considering the different total number of visits between the years, in the second pandemic year, the proportion of visits related to mental health problems was higher, 6.4% vs. 5.6%, but not statistically different from the pre-pandemic year, *p* = 0.07.

**Table 1:** Main features of adolescents’ PED visits during the pre-pandemic, the first and the second year of the COVID-19 pandemic.

	2019/2020 (*n* 26,019)	2020/2021 (*n* 12,704)	2021/2022 (*n* 19,465)	% Change between 19/20 and 20/21	% Change between 19/20 and 21/22	% Change between 20/21 and 21/22	*p* value between 19/20 and 20/21	*p* value between 19/20 and 21/22	*p* value between 20/21 and 21/22
Number of PED visits of adolescents, *n* (%)	5,501 (21.2)	3,163 (24.9)	4,746 (24.38)	−43%	−14%	+50%	<0.0001	<0.0001	0.29
Age, years, median (IQR)	15 (14–16)	15 (14–16)	15 (14–16)	-	-	-	0.002	0.01	0.50
Females, *n* (%)	2,479 (45.1)	1,339 (42.3)	2,074 (43.7)	−46%	−16%	+55%	0.01	0.17	0.23
Triage code, *n* (%):									
Non-urgent	1,723 (31.3)	1,091 (34.5)	1,648 (34.7)	−37%	−4%	+51%	0.002	0.0003	0.83
Minor urgencies	3,213 (58.4)	1,779 (56.2)	2,604 (54.9)	−45%	−19%	+46%	0.05	0.0003	0.23
Urgencies	553 (10.1)	281 (8.9)	481 (10.1)	−49%	−13%	+71%	0.08	0.89	0.07
Emergencies/resuscitation	12 (0.2)	12 (0.4)	13 (0.3)	0%	+8%	+8%	0.17	0.57	0.41
Discharge diagnosis, *n* (%):									
Injuries	2,533 (46.1)	1,321 (41.8)	2,357 (49.7)	−48%	−7%	+78%	0.0001	0.0003	<0.0001
Infectious diseases	1,260 (22.9)	771 (24.4)	777 (16.4)	−39%	−38%	+0.7%	0.12	<0.0001	<0.0001
Mental health problems	306 (5.5)	172 (5.4)	304 (6.4)	−44%	−0.7%	+77%	0.81	0.07	0.08
Neurological diseases	233 (4.2)	137 (4.3)	262 (5.5)	−41%	+12%	+91%	0.83	0.003	0.02
Surgical diseases	108 (2.0)	97 (3.1)	87 (1.8)	−10%	−19%	−1%	0.001	0.63	0.0004
Other	1,061 (19.3)	665 (21.0)	959 (20.2)	−37%	−10%	+44%	0.05	0.24	0.38
Hospital admissions, *n* (%)	199 (3.6)	180 (5.7)	244 (5.1)	−10%	+23%	+36%	<0.0001	0.0002	0.29
Ward of destination of admitted patients, *n* (%):									
Pediatric ward	51 (25.6)	49 (27.2)	33 (13.5)	−4%	−35%	−33%	0.73	0.001	0.0004
Surgery ward	47 (23.6)	57 (31.7)	66 (27.1)	+21%	+40%	+16%	0.08	0.41	0.30
Neuro-psychiatric ward	43 (21.6)	32 (17.8)	51 (20.9)	−26%	+19%	+59%	0.35	0.86	0.42
Orthopedic ward	38 (19.1)	21 (11.7)	40 (16.4)	−45%	+5%	+90%	0.05	0.46	0.17
Intensive care unit	0 (0.0)	5 (2.8)	4 (1.6)	+500%	+400%	−20%	0.02*	0.13*	0.50*
Other	20 (10.1)	16 (8.9)	50 (20.5)	−20%	+150%	+212%	0.70	0.003	0.001

*Fisher's exact test.

In the second pandemic year more adolescents were hospitalized compared to the pre-pandemic year: 244 (5.1%) vs. 199 (3.6%), +23%, *p* = 0.0002.

**Figure 1:**
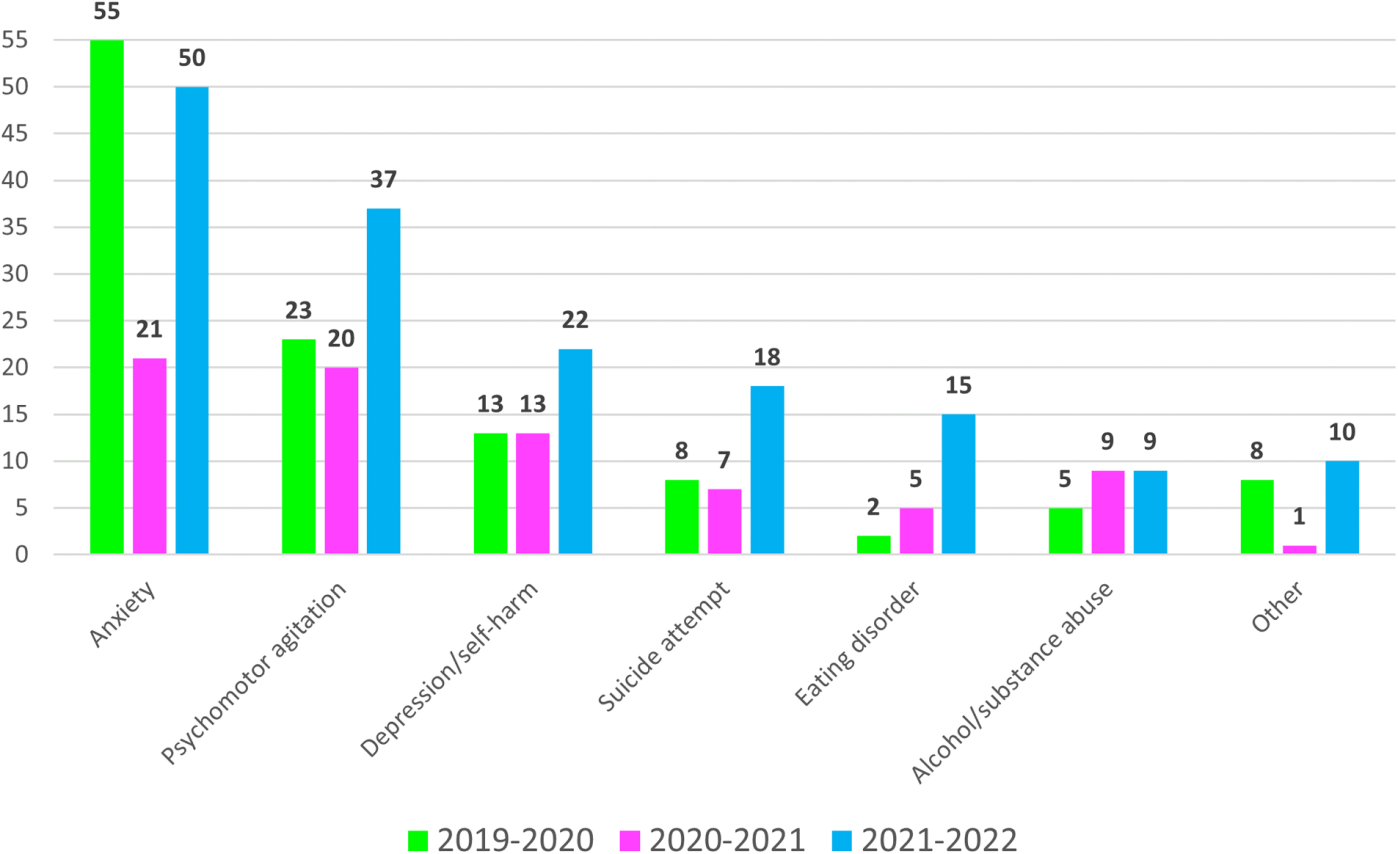
Number of urgent psychiatric consultations during the study period detailed for every mental health diagnosis.

The Table 2 shows the characteristics of the patients who accessed the PED with mental health problems during the study period. During the study period, 782 adolescents (5.8%) received a diagnosis related to mental health problems, 306 (5.5%) in the pre-pandemic year, 172 (5.4%) in the first pandemic year, and 304 (6.4%) in the second pandemic year. The prevalence of females was significantly higher 220 (72.4%) vs. 197 (64.4%), +12%, *p* = 0.03. The level of triage priority of the visits was not statistically different. Nevertheless, in the second year of the pandemic more adolescents were admitted, 44 (14.5%) vs. 34 (11.1%), +29%, *p* = 0.21, and more urgent psychiatric consultations were needed, 161 (53%) vs. 114 (37.3%), +41%, *p* < 0.0001. The Figure 1 indicates the number of urgent psychiatric consultations for every mental health diagnosis in the study period. The Table 2 shows also the distribution among the years of the different diagnoses received by these patients. Subjects with anxiety decreased in the first year of the pandemic by 54% and rose again in the second year of the pandemic. Agitated patients revealed a similar distribution. On the contrary, the number of subjects with depression/self-harm, suicide attempts, and eating disorders was stable in the first year of the pandemic but sharply grew in the second one. The number of patients with depression/self-harm was 31 (10.2%) vs. 18 (5.9%), +72%, *p* = 0.05. The number of suicide attempts was 23 (7.5%) vs. 8 (2.6%), +188%, *p* = 0.01. The total of cases with eating disorders was 21 (6.9%) vs. 5 (1.6%), +320%, *p* = 0.001. Finally, in the pandemic years, the number of adolescents presenting for alcohol or substance abuse remained lower compared to the pre-pandemic year, 33 (19.1%) and 47 (15.4%) vs. 75 (24.5%) cases, respectively, −56% and −37%, *p* = 0.18 and *p* = 0.01.

**Table 2:** Main features of the adolescents who accessed the PED with mental health problems during the study period.

PED visits of adolescents with mental health problems	2019/2020 (*n* 306)	2020/2021 (*n* 172)	2021/2022 (*n* 304)	% Change between 19/20 and 20/21	% Change between 19/20 and 21/22	% Change between 20/21 and 21/22	*p* value between 19/20 and 20/21	*p* value between 19/20 and 21/22	*p* value between 20/21 and 21/22
Age, years, median (IQR)	16 (14–17)	16 (15–17)	16 (14–17)	-	-	-	0.59	0.93	0.66
Females, *n* (%)	197 (64.4)	104 (60.5)	220 (72.4)	−47%	+12%	+112%	0.40	0.03	0.01
Triage code, *n* (%):									
Non-urgent	52 (17.0)	32 (18.6)	52 (17.1)	−38%	0%	+63%	0.66	0.97	0.68
Minor urgencies	126 (41.2)	85 (49.4)	145 (47.7)	−33%	+15%	+71%	0.08	0.11	0.72
Urgencies	122 (39.9)	52 (30.2)	104 (34.2)	−57%	−15%	+100%	0.04	0.15	0.37
Emergencies/resuscitation	6 (2.0)	3 (1.7)	3 (1.0)	−50%	−50%	0%	1.00*	0.51*	0.67*
PED diagnosis, *n* (%):									
Anxiety	141 (46.1)	65 (37.8)	122 (40.1)	−54%	−13%	+88%	0.08	0.14	0.62
Psychomotor agitation	46 (15.0)	30 (17.4)	47 (15.5)	−35%	+2%	+57%	0.49	0.88	0.57
Depression/self-harm	18 (5.9)	18 (10.5)	31 (10.2)	0%	+72%	+72%	0.07	0.05	0.93
Suicide attempt	8 (2.6)	8 (4.7)	23 (7.6)	0%	+188%	+188%	0.24	0.01	0.22
Eating disorder	5 (1.6)	8 (4.7)	21 (6.9)	+60%	+320%	+162%	0.05	0.001	0.32
Alcohol/substance abuse	75 (24.5)	33 (19.2)	47 (15.5)	−56%	−37%	+42%	0.18	0.01	0.30
Other	13 (4.3)	10 (5.8)	13 (4.3)	−23%	0%	+30%	0.44	0.99	0.45
Urgent psychiatric consultations, *n* (%)	114 (37.3)	76 (44.2)	161 (53.0)	−38%	+41%	+112%	0.14	<0.0001	0.07
Hospital admissions, *n* (%)	34 (11.1)	22 (12.8)	44 (14.5)	−35%	+29%	+100%	0.58	0.21	0.61

*Fisher's exact test.

## Discussion

This study described a drastic increase in the cases of suicide attempts, self-harm, and eating disorders among the adolescents who presented at the PED during the second year of the COVID-19 pandemic. This growth was astonishing compared to the pre-pandemic year and the first year of the pandemic: +72% of cases of self-harm, +188% of cases of suicide attempts, and +320% of cases of eating disorders overall. We did not see substantial growth in mental health problems in general. The number of cases that arrived in the second pandemic year was equal compared to the pre-pandemic year. However, these latter cases were clinically more severe. More adolescents received inpatient care, and more urgent psychiatric consultations were required.

The more time passed, the more the international evidence highlighted the detrimental psychological effects and the considerable burden of psychological suffering among children and adolescents related to forced isolation, school closure, the ban of sports, and social activities, and the pandemic in general. A meta-analysis of 29 studies, covering 80,879 subjects, reported that 25% of children and adolescents were experiencing depressive symptoms globally (6). The same study revealed a high percentage of anxiety, 20%, with rates doubling the pre-pandemic levels.

A systematic review and meta-analysis of studies describing suicidal ideation, suicide attempts, and suicide, covering 120,000 participants worldwide, mainly adults, in the early phase of the COVID-19 pandemic showed a prevalence of suicidal ideation of 12.1% (CI 9.3–15.2), higher than the pre-pandemic data (7). On the contrary, a cross-sectional study, specifically focused on adolescents accessing the emergency department for suicidal thoughts and behaviours in Northern Carolina, USA, showed a similar number of cases in the pre-pandemic and in the first pandemic year (8). A slightly augmented prevalence of psychiatric hospital admissions related to suicide attempts and eating disorders was already described in Italy in the first pandemic year compared to the pre-pandemic year (9). Our study underlined how this phenomenon intensified in the second pandemic year. In the same way, a multicenter German study demonstrated a 2.84 increased risk of intensive care unit admission for suicide attempts, in adolescents 12–17 years of age, in the second year of the pandemic compared to the years before (10).

The emergence of eating disorders among adolescents was described immediately after the first phases of rigid lockdown put in place in many countries to try to stop the contagion spread (11, 12) and was reported as sustained 10 months after the pandemic's beginning (13). In our setting, this abrupt increase in cases was evident in the second pandemic year.

Several previous studies described how the early phases of this pandemic influenced the psychological well-being of children and young people (14). This study highlighted how longer exposure to the stressors associated to the pandemic led to ongoing detrimental psychological effects. In many cases, social isolation, family financial difficulties, missed opportunities of growth and development remained after the relaxation of rigid lockdowns and this cumulative effect impacted on the mental health of children and adolescents.

Moreover, during the second pandemic year, most schools reopened and thus stressors, such as academic difficulties and bullying, started to affect children and adolescents again.

It has been reported that self-rated psychological problems, functional impairment, depression, and referral to psychiatric emergency services, appeared higher among young people after school re-openings (15, 16).

In this study, we observed an increase of the female predominance among subjects with mental health symptoms during the second year of the pandemic and this could be explained by the increase of specific mental health problems associated with a strong female predominance such as eating disorders.

Lockdowns and social distancing measures completely changed the epidemiology of pediatric emergency department visits (17, 18). This study had the value of offering a complete picture of the PED visits during the first and the second years of the pandemic compared to the pre-pandemic year and framing the distribution of mental health presentations from the broader perspective of PED presentations. In our population, the prevalence of infectious diseases among adolescents has not yet returned to pre-pandemic levels. In the first and second years of the pandemic it was substantially stable. Conversely, the relaxation of the social distancing measures that occurred in Italy's second year of the pandemic was associated with a return to a similar prevalence of injuries to the pre-pandemic year. While the crude number of mental health presentations also returned to pre-pandemic levels, we highlighted how the causes and the complexity of the cases differed. Beside the pandemic and the measures putted in place to limit its spread, several other possible confounders may have influenced our results. Nevertheless, in our comments we preferred to remain strictly in line with our results avoiding to suggest other possible speculative explanations. The emergency department has been reported to be the first health care contact for most children and adolescents with mental health problems (19). Again, our departments should be prepared for the best possible support.

This study has several limitations. Due to its retrospective design, some cases may have been missed or mislabeled. The number of non-consenting caregivers in our Institution is exceptional. Nevertheless, we are not able to provide the exact number of non-consenting cases. We considered only the last pre-pandemic year, so we could not describe the trend of PED visits for mental health problems over an extended period of time. Therefore, we can't exclude that some data were part of a normal change in PED flows. Moreover, we can't exclude the influence of other potential confounders on the results. Being a single-center experience, the generalization of the results is limited. Our data refer to the adolescents who have accessed the PED only, so we cannot estimate the actual prevalence of mental health problems in our general population of adolescents. Moreover, in our territory, primary care physicians may refer children and adolescents directly to mental health services, so we can presume that the population of subjects involved was larger than what we described in this manuscript.

We cannot provide data for late adolescents, 18–25 years of age, because access to our PED is limited to patients aged 0–17. Our Institute is the only one in the region with a psychiatric ward, so we cannot exclude that some patients could have accessed our PED aiming for inpatient psychiatric care. Finally, only some adolescents who received a PED diagnosis of mental health problems were evaluated by a child psychiatrist.

## Conclusion

This retrospective study showed that pediatric emergency department visits related to suicide attempts and eating disorders in adolescents had the most significant increase in the second year of the pandemic compared both to the first and the pre-pandemic one. Multicenter studies should confirm our findings.

## Data Availability

The raw data supporting the conclusions of this article will be made available by the authors, without undue reservation.
